# Primary Care-Based Estimates of Influenza Vaccine Effectiveness in Hungary, 2024/25

**DOI:** 10.3390/vaccines14040342

**Published:** 2026-04-13

**Authors:** Gergő Túri, Viktória Velkey, Krisztina Mucsányiné Juhász, Katalin Krisztalovics, Annamária Ferenczi, Csaba Luca, Edit Bilics, Katalin Kristóf, Beatrix Oroszi

**Affiliations:** 1 National Laboratory for Health Security, Epidemiology and Surveillance Centre, Semmelweis University, 1085 Budapest, Hungary; 2 Doctoral School of Health Sciences, University of Debrecen, 4032 Debrecen, Hungary; 3 Clinical Microbiology Laboratory, Institute of Laboratory Medicine, Semmelweis University, 1085 Budapest, Hungary

**Keywords:** vaccine, effectiveness, test-negative design, influenza, primary care, Hungary, Europe

## Abstract

Background: The 2024/25 influenza season in Hungary experienced a major surge in cases, the largest since the COVID-19 pandemic. We evaluated influenza vaccine effectiveness (VE) in primary care settings among adults and vaccination target groups, and also according to time since vaccination, prior seasonal vaccination, and influenza type. Methods: A test-negative case–control study was conducted in Hungary. Data and specimens were collected from primary care patients with an acute respiratory infection (ARI). Patients with positive PCR test results for influenza were classified as cases, while those with negative test results for influenza were classified as controls. Adjusted VEs were calculated using logistic regression as (1 − odds ratio of vaccination) × 100. Results: Between November 2024 and May 2025, 2074 patients were included in the analysis, of whom 395 cases had influenza. Of the 129 vaccinated patients, 123 (95%) received trivalent inactivated adjuvanted whole-cell vaccine (TIAV), and 6 (5%) received quadrivalent split-virion vaccine. The VE against any influenza was 53% (95% CI: 13–74) in the 18+ age group and 52% (95% CI: 7–75) in the target group for vaccination. The VE against any influenza was 63% (95% CI: 17–84) 14–89 days after vaccination, and 27% (95% CI: −67–68) 90 days or more after vaccination. The VE against any influenza was 56% (95% CI: 1–80) with both current and prior seasonal vaccination, and 5% (95% CI: −64–45) with only prior seasonal vaccination. The VE against influenza A was 39% (95% CI: −16–68), and against influenza B was 80% (95% CI: 2–96). Conclusions: We observed moderate vaccine effectiveness against any influenza, with higher protection within three months after vaccination. Our research findings provide evidence to inform the development of vaccines and the scheduling of vaccination campaigns, with the aim of maximizing the level of protection provided by vaccines throughout the entire influenza season.

## 1. Introduction

The influenza season of 2024/25 in Hungary was characterized by a substantial surge in influenza cases, representing the largest epidemic in the country since the onset of the COVID-19 pandemic. It has been reported that there were elevated levels of influenza activity between January and March 2025, which were accompanied by significant excess mortality among individuals aged 65 and over from week 52 of 2024 to week 9 of 2025 [1,2]. Alongside this, the 2024/2025 respiratory season witnessed the lowest recorded influenza vaccination rates in Hungary over the past two decades among the elderly population [3].

Influenza vaccination represents a fundamental public health instrument for preventing influenza-related illnesses as well as the mitigation of the complications from the disease. In Hungary, the 2024/25 influenza vaccination campaign for the respiratory season commenced in week 42 of 2024, with the following groups being targeted: individuals aged 60 and over, those with chronic diseases, and pregnant women. During the 2024/25 season, a trivalent egg-based inactivated adjuvanted whole-cell vaccine (TIAV) was provided free of charge to this target group [4]. A quadrivalent split-virion vaccine was also available for a charge [5]. The administration of influenza vaccinations was carried out by medical doctors and nurses in general practitioner (GP) practices and vaccination centers. The effectiveness of the Hungarian vaccination program is indicated by the decline in vaccination coverage over the years, which, in the 2024/25 respiratory season, did not reach 20% among the elderly population [3].

Since 2021, the Epidemiology and Surveillance Centre of Semmelweis University has been operating a novel respiratory surveillance system as part of the Vaccine Effectiveness, Burden and Impact Studies (VEBIS) network [6,7,8,9,10]. This surveillance system is innovative within the Hungarian context due to its case-based data collection and sequencing of SARS-CoV-2 and influenza-positive samples [10]. The surveillance system comprises more than 100 participating general practitioners who actively recruit patients and collect data related to acute respiratory infections on a year-round basis. These practitioners adhere to a standardized methodological protocol.

Influenza epidemics are a recurring problem on an annual basis, and enhancing the effectiveness of prevention programs remains a challenge. Continuous monitoring of the influenza VE is crucial for evaluating vaccination programs and strategies, and for the establishment of vaccination recommendations for the forthcoming respiratory season. This is of particular importance, given the substantial variability of influenza VE, which is subject to change depending on the population, season, and circulating virus subtype [11]. Furthermore, influenza VE estimates may be utilized to formulate public health messages that emphasize the importance of ensuring higher vaccine uptake. The influenza VE by time since vaccination may be used to inform the timing of existing vaccination programs, as several studies have demonstrated a gradual decline in protection over time [12,13]. Better understanding of declining influenza VE across diverse populations is of importance because of the early administration of vaccinations, often several months prior to the peak circulation of influenza in numerous countries, including Hungary. Furthermore, the influenza VE analysis by seasonal vaccination status may add to the evidence that indicated that repeated vaccination in consecutive years may affect current influenza VE [14,15].

This study aims to estimate influenza vaccine effectiveness in the 2024/25 respiratory season in Hungary against any influenza in the 18+ age group and in the target group for vaccination; according to time since vaccination; by seasonal vaccination status; and by influenza types.

## 2. Materials and Methods

### 2.1. Study Design and Study Population

We conducted a primary care-based test-negative design study involving 110 Hungarian GPs. The analysis period was defined from the onset of symptoms of the first laboratory-confirmed influenza case (week 45 of 2024) through week 22 of 2025. The Hungarian study protocol was adapted from the European Centre for Disease Prevention and Control (ECDC)’s core protocol for vaccine effectiveness studies regarding symptomatic, laboratory-confirmed influenza infection in primary care settings [16]. The study was conducted as part of the VEBIS project [16].

Patients aged 18 years or older were enrolled in the study if they presented to one of the general practitioners with sudden-onset symptoms meeting the European Union definition of acute respiratory infection (EU ARI). This definition includes at least one of the following respiratory symptoms: sore throat, cough, coryza, shortness of breath, and a GP’s judgment that the illness was due to an infection [16]. All patients who met the case definition were invited to participate in the study. After giving their informed consent, participants had respiratory samples taken by their GPs for testing. Those who did not receive the influenza vaccine were included in the reference group for the analysis. We systematically recorded the weekly recruitment numbers for both cases and controls based on the date of swabbing. Patients were consecutively recruited based on predefined inclusion criteria, and no matching was applied at enrolment; potential confounders were addressed through adjustment in the analysis. Furthermore, we documented the number of confirmed influenza cases, classified by type and subtype, along with the characteristics of the case and control groups.

### 2.2. Data Collection

The participating GPs collected basic demographic data, symptoms, underlying conditions, and current and previous respiratory season influenza vaccination status using RedCap (Research Electronic Data Capture, Vanderbilt University, TN, USA) version 15.5.1, a web-based software platform designed for research data collection and management [17]. The primary sources of information on influenza vaccination status were general practitioner records or the National eHealth Infrastructure, with less reliance on self-reporting.

### 2.3. Patient Exclusion Criteria

Patients who resided in a residential care facility, had taken antiviral medication within 14 days prior to sampling, had contraindications to influenza vaccination, had no information available on their influenza vaccination status, swabbed more than 7 days after the onset of symptoms, or had conflicting PCR test results (previous positive and current negative influenza PCR test results related to the present symptoms) were excluded from the study.

### 2.4. Vaccination Definition

Participants were considered vaccinated 14 days after receiving a seasonal influenza vaccine during the 2024/25 season. Participants who did not receive an influenza vaccine during the current respiratory season were considered unvaccinated. Participants who received any influenza vaccine during the previous season (from week 40, 2023 to week 20, 2024) were considered vaccinated during the previous season. Participants who received the influenza vaccine in both the 2023/24 and 2024/25 seasons were considered vaccinated in both seasons.

### 2.5. Statistical Analysis

Data analysis was performed using the Stata 17.0 BE-Basic Edition (College Station, TX, USA) statistical program. Patients who tested positive for influenza by PCR laboratory testing were considered influenza cases, while those who tested negative for influenza by PCR were classified as the control group.

Logistic regression was used with influenza case status as the outcome and influenza vaccination status as the exposure. The model was adjusted for age, sex, the presence of at least one chronic disease, and the month of symptom onset. The influenza VE was calculated using the formula of (1 − odds ratio of vaccination) × 100 (%), using unvaccinated patients as the reference group. Because of small sample size and sparse data in some strata, Firth’s penalized logistic regression was used for stratified analysis to reduce small-sample bias and address potential separation. The influenza VE was also evaluated among the vaccination target groups, as well as by time since vaccination (14–89 days and 90+ days) and influenza type (influenza A and influenza B). Furthermore, we estimated influenza VE among those vaccinated in both respiratory seasons. Subgroup VE estimates were only reported when data size allowed reasonable estimation.

We analyzed whether rapid antigen tests (RATs) prior to GP consultation could influence influenza VE estimates by differentially affecting GP consultations. We collected information on previous RAT usage related to current symptoms, specifying the type of test (RAT, PCR, or both) and the pathogen(s) tested for. During the analysis, we included the variable of RAT use prior to the GP consultation in the logistic regression model. Age was included as a categorical variable with two groups: 18–59 years and 60 years or older. The analysis period for influenza B was defined from the onset of symptoms of the first laboratory-confirmed influenza B case (week 51 of 2024) through week 22 of 2025.

## 3. Results

### 3.1. Study Population and Descriptive Analysis

**Figure 1:**
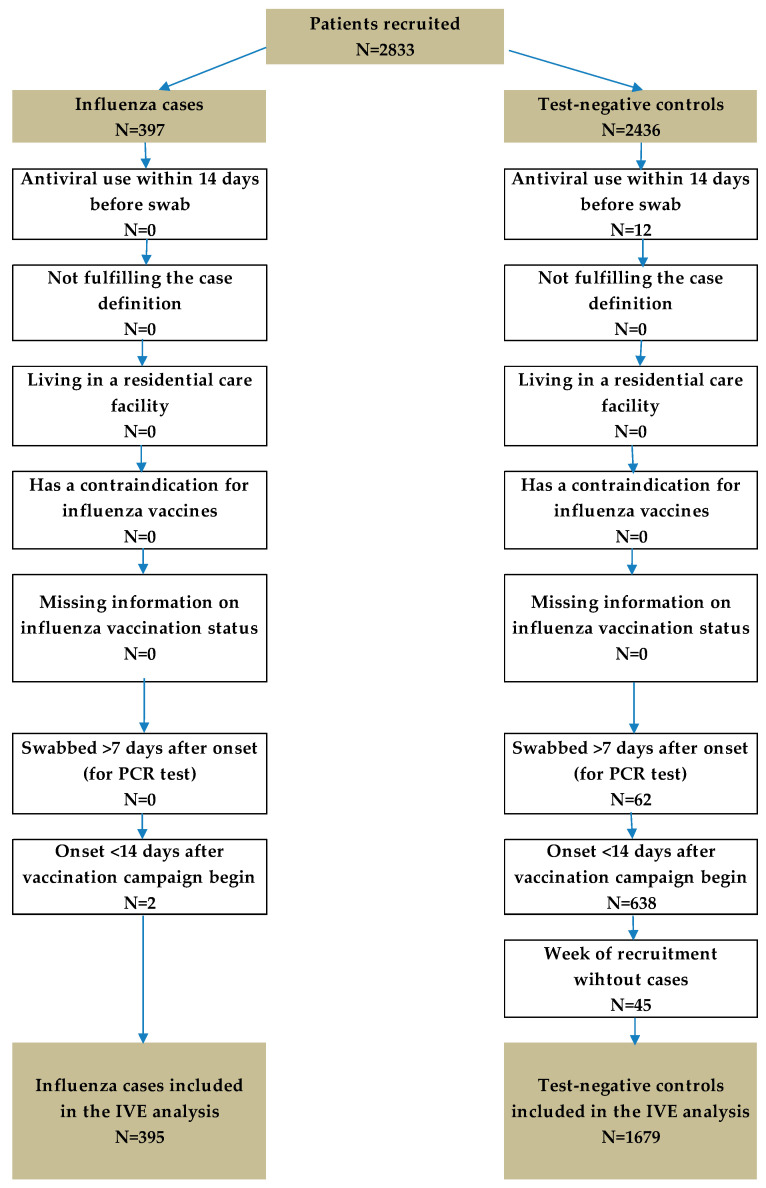
Study eligibility flowchart, primary care-based influenza VE study, Hungary, August 2024–May 2025.

The recruitment period was from 23 August 2024 to 30 May 2025, during which a total of 2833 patients were enrolled in the study. In accordance with the exclusion criteria outlined in the protocol, 759 patients (2 cases and 757 controls) were excluded prior to analysis. The exclusions flow chart is summarized in Figure 1.

**Figure 2:**
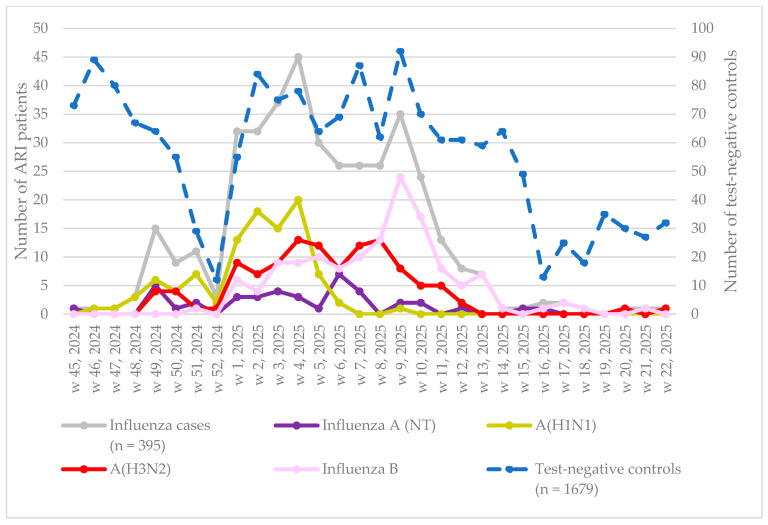
Influenza cases and test-negative controls included in the analysis, by week of onset, and the corresponding number of influenza types and subtypes in the primary care-based influenza VE study, Hungary, November 2024–May 2025.

A total of 2074 patients were included in the influenza VE analysis, comprising 395 cases and 1679 controls (Figure 2). During the recruitment period, a total of 395 influenza-positive samples were typed as part of the study. Of these, 41 (10%) were influenza A not-subtyped (NT), 100 (25%) were influenza A (H1N1), 115 (29%) were influenza A (H3N2), 137 (35%) were influenza B, and 2 (1%) were influenza A+B. Influenza A (H1N1) was predominant between week 45 of 2024 and week 4 of 2025, influenza A (H3N2) between weeks 5 and 7 of 2025, and influenza B between weeks 8 and 22 of 2025.

Among the participants eligible for inclusion in the study, the median age was slightly lower in cases (42 years, interquartile range [IQR]: 32–53) than in controls (44 years, IQR: 31–56) (Table 1). The proportion of women was slightly lower among cases (53%) than among controls (58%). The distribution of age groups was found to be similar among cases (18–64 years: 83%; 60+ years: 17%) and controls (18–64 years: 82%; 60+ years: 18%). In both cases and controls, 29% reported living with at least one chronic illness.

Vaccination status was ascertained based on electronic health records of general practitioners (77%), the National Immunization Registry (17%), and self-reporting (6%). During the 2024/2025 respiratory season, the proportion of cases and controls who received influenza vaccination was 4% and 7%, respectively. Of the 129 vaccinated patients, 123 (95%) received a TIAV and 6 (5%) received a quadrivalent split-virion vaccine.

### 3.2. Vaccine Effectiveness Estimates Against Any Influenza Amongst Patients Aged 18 Years Old and Over, and in the Target Groups

Amongst patients aged 18 and over, the adjusted influenza VE was 53% (95% confidence interval [CI]: 13–74), and 52% (95% CI: 7–75) in the target group for vaccination (Table 2). Adding the variable of RAT use in the model did not change the results (Supplementary Table S1). In addition, no significant association was observed between influenza vaccination status and performing a RAT prior to the GP consultation (OR = 1.10, 95% CI: 0.56–2.15). As a sensitivity analysis, restricting the study to TIAV recipients yielded VE estimates comparable to the main analysis (Supplementary Table S2). In addition, no significant association was observed between influenza vaccination status and the number of GP consultations in the previous 365 days (OR = 1.05, 95% CI: 0.73–1.52), nor with hospitalizations in the previous 365 days (OR = 0.91, 95% CI: 0.66–1.82). Furthermore, no significant association was observed between influenza case status and the number of GP consultations in the previous 365 days (OR = 1.23, 95% CI: 0.82–2.03), nor with hospitalizations in the previous 365 days (OR = 0.88, 95% CI: 0.59–1.91).

### 3.3. Vaccine Effectiveness Estimates Against Any Influenza Amongst Patients Aged 18 Years Old and Over, According to Time Since Vaccination

The adjusted influenza VE was higher 14–89 days after vaccination (63%; 95% CI: 17–84%) than 90+ days since vaccination (27%; 95% CI: −67–68%) (Table 3).

### 3.4. Vaccine Effectiveness Estimates Against Any Influenza Amongst Patients Aged 18 Years Old and Over, by Seasonal Vaccination Status

For individuals 18 years and older, the adjusted influenza VE for those with both current and prior season vaccination was 56% (95% CI: 1–80%), and 5% (95% CI: −64–45%) for those with only prior season vaccination (Table 4).

### 3.5. Vaccine Effectiveness Estimates Against Influenza Amongst Patients Aged 18 Years Old and Over, by Influenza Types

For individuals aged 18 years and over, the adjusted VE against influenza A was 39% (95% CI: −16–68%), and 80% (95% CI: 2–96%) against influenza B (Table 5).

## 4. Discussion

Following the conclusion of the 2024/25 respiratory season in Hungary, we estimated 53% (95% CI: 13–74) vaccine effectiveness against any influenza in individuals aged 18 years and over, and a 52% vaccine effectiveness (95% CI: 7–75) in the target group of the vaccination program.

These end-of-season estimates are consistent with the interim pooled influenza VE estimates of eight studies, involving 17 European countries, which reported a pooled influenza VE of 40–53% among individuals aged 18 years old and over, and 31–55% among the target group for vaccination [18]. As stated in the mid-season report from France, the influenza VE was 42% (95% CI: 37–46), a figure which is slightly lower than the results obtained in this study. A report from Germany documented an even lower influenza VE of 31% (95% CI: 1–52) across all age groups [19,20]. The findings of the present study are consistent with the VEBIS network’s overall influenza VE estimates for the preceding two respiratory seasons in primary care settings (51–53%) and for the target group for vaccination (45–53%) [8,21]. In the 2024/2025 respiratory season the TIAV was the most prevalently used vaccine in Hungary.

A decline in the effectiveness of vaccines has frequently been documented in the context of SARS-CoV-2, often occurring within a few months following administration. In contrast, the within-season decline in the effectiveness of influenza vaccines has received comparatively less attention in recent years. The existing study results are controversial, which draws attention to the fact that the phenomenon of declining vaccine effectiveness is not yet fully understood. A systematic review and meta-analysis found evidence of persistent antibody responses following influenza vaccination over the course of a typical influenza season [22]. Conversely, another review found evidence that HI antibody responses following influenza vaccination do not consistently persist throughout the year in older adults [23]. The inherent limitations of such studies are evident in the context of measuring antibody titers, which, despite their importance, are not a perfect indicator of declining protection [22,23,24].

The findings of the present study, which utilized laboratory-confirmed influenza as an outcome, indicate that the vaccine exhibited a high degree of effectiveness (63%; 95% CI: 17–84%) against any influenza in the 18+ age group within 14–89 days following vaccine administration. However, the VE was lower (27%; 95% CI: −67–68) 90+ days after vaccination. Although no universally accepted cut-off exists for categorizing time since influenza vaccination, the applied intervals were informed by prior test-negative design studies, which commonly distinguish VE estimates before and after approximately three months post-vaccination to assess within-season waning [25,26]. A French study conducted in primary care settings during the 2023/2024 respiratory season also reported similar findings, estimating an overall influenza VE of (51%; 95% CI: 45–56) between 15 and 90 days after vaccination, but only 35% (95% CI: 24–45) between 91 and 180 days after vaccination [25]. In the preceding years, from 2010 to 2017, an English study reported an influenza VE of 44.7% (95% CI: 21.2 to 61.2) within 14 to 90 days following vaccination, while only 14.5% (95% CI: −18.2 to 38.2) was observed 90+ days after vaccination [26]. Concurrently, the I-MOVE, an analysis of respiratory seasons from 2010 to 2015, also demonstrated within-season decline in the influenza VE [27].

Test-negative design studies to evaluate the intra-seasonal decrease in influenza VE are also not without limitations. Firstly, it is not possible to separate waning immunity from the effect of an emergent antigenically drifted virus within the influenza season. This is because such a virus can evade antibodies from previous infections or earlier vaccinations. Secondly, the depletion of susceptible individuals can distort effectiveness estimates by making the remaining unvaccinated population appear healthier than they are, as those who were more vulnerable may have already been infected [12,28]. These biases have the potential to result in either an over- or underestimation of a vaccine’s effectiveness.

Despite the absence of a universally accepted methodology for the assessment of waning immunity, an increasing amount of evidence points to within-season decline in influenza VE. Optimizing the protection provided by seasonal influenza vaccines within a given season is a pressing issue, given the potential for significant delays between vaccine administration and exposure to influenza. As demonstrated by the case of Hungary, a considerable time interval can elapse between the initiation of the vaccination campaign and the subsequent surge in influenza cases. This interval presents a good opportunity for the observation of waning vaccine effectiveness. International modeling studies have investigated the potential benefits of bringing the vaccination program closer to the start of the influenza season, with a view to the vaccine providing greater protection [29,30]. It is suggested that, in light of the epidemiological data and the findings of the modelling studies, the potential merits of rescheduling vaccination campaigns should be given due consideration. The objective of such a strategy would be to optimize the level of protection provided by vaccines throughout the entire influenza season.

We observed moderate influenza VE (56%; 95% CI: 1–80%) among individuals who received vaccination in both the current and previous seasons. In contrast, the results do not confirm a significant protective effect for the vaccine administered only in the previous year against symptomatic illness in the current season (VE: 5%; 95% CI: −64–45%). The existing literature on this issue is inconsistent. For example, a Spanish study conducted in a primary care setting found that vaccination in previous seasons provided some protection against influenza [31]. However, a further outcome of a systematic review and meta-analysis aligns with our results, namely that vaccination exclusively in the previous season offers negligible or minimal protection against symptomatic illness in the current season [15]. Following the evaluation of the available data, there is sufficient evidence that justifies the continued recommendation of annual influenza vaccinations for the general population.

According to the WHO recommendation for the 2024/25 northern hemisphere influenza season, the vaccine composition included A/Victoria/4897/2022 (H1N1)pdm09-like viruses (clade 6B.1A.5a.2a), A/Thailand/8/2022 (H3N2)-like viruses (clade 3C.2a1b.2a.2), and B/Austria/1359417/2021-like viruses (B/Victoria lineage, clade V1A.3a.2) [32]. In comparison, circulating viruses in Europe during the 2024/25 season largely belonged to related but genetically diverse subclades, with A(H1N1)pdm09 viruses predominantly from clade 5a.2a (C.1.9), A(H3N2) viruses from subclade 2a.3a.1 (J.2), and influenza B viruses from subclade V1A.3a.2 (C.5.1), indicating overall antigenic similarity but ongoing genetic diversification that may have influenced subtype-specific VE estimates [33].

It is important to note that epidemiological studies of test-negative design are observational studies. Consequently, they are susceptible to bias and confounding. The impact of potential confounding factors should be monitored wherever possible. The utilization of RATs for the identification of influenza and SARS-CoV-2 may have an impact on visits to general practitioners, which could potentially result in patient selection and subsequent bias in the results of the influenza VE estimates [34,35,36]. In the present study, 6.5% of patients admitted to using some form of RATs prior to consulting their GP, with 5% of all patients using only a test for SARS-CoV-2, 1.5% of all patients using both a test for SARS-CoV-2 and an influenza, and 0.5% of all patients using only an influenza RAT. The study found no association between vaccination status and previous rapid testing, and no evidence that RAT use before GP consultation was a confounding factor. It is important to note that public habits regarding the use of RAT may be subject to change on an annual basis. Therefore, it is advisable to continue the collection of information on rapid test use and conduct in-depth analyses to check for potential bias or confounding effects in the forthcoming seasons [34,35,36].

In addition, confounding due to unmeasured factors cannot be excluded. Information on prior influenza infection history and socioeconomic status was not available in our dataset and therefore could not be accounted for in the analysis. Although healthcare-seeking behavior may influence inclusion in test-negative design studies, we found no evidence of an association between proxy indicators available in our dataset (number of GP consultations and hospitalizations in the previous 365 days) and either influenza vaccination status or influenza case status, suggesting that their confounding effect is likely limited. Nevertheless, these factors may have influenced the VE estimates to some extent [37,38,39].

Despite the continuous expansion of our research network in recent years, a significant limitation of our study remains the inadequate sample size. This limitation results in broad confidence intervals surrounding certain point estimates, precluding the execution of further age- or influenza virus subtype-specific stratified VE analyses. The low number of vaccinated individuals, particularly among cases, limited the precision of some VE estimates and resulted in wide confidence intervals in several stratified analyses [40,41].

## 5. Conclusions

The Hungarian study provides a valuable example of how a sustainable GP-based research network for respiratory viruses can be operationalized at the national level. Such research networks may facilitate the evaluation and planning of national vaccination programs. Additionally, they contribute to international programs and studies through pooled analysis of diverse populations. This study from the 2024/25 respiratory season indicated moderate vaccine effectiveness against any influenza, with higher levels of protection observed within three months after vaccination. The findings of the research provide evidence to inform the development of vaccines and the scheduling of vaccination campaigns, with the aim of maximizing the level of protection provided by vaccines throughout the entire influenza season. Influenza vaccination was associated with moderate protection, with an estimated vaccine effectiveness of around 50% against laboratory-confirmed influenza in the 2024/25 season in Hungary. This finding serves to emphasize that vaccination remains the most effective method of protection against influenza. Given the suboptimal performance of the influenza prevention program in Hungary, the results of this study may provide important arguments to inform a communication campaign aimed at increasing vaccine acceptance among the target group for vaccination. Ultimately, the results obtained from this national research network may be utilized in the formulation of data-driven prevention program plans, encompassing initiatives designed to enhance pandemic preparedness.

## Figures and Tables

**Table 1:** Baseline characteristics of influenza cases (*n* = 395) and test-negative controls (*n* = 1679) in the primary care-based influenza VE study, Hungary, November 2024–May 2025.

Characteristic	Influenza Cases	Test-Negative Controls
	(*n* = 395)	(*n* = 1679)
Age, median [IQR]	42 [32–53]	44 [31–56]
**Age group (years), *n* (%)**		
18–59	327 (83)	1373 (82)
60+	68 (17)	306 (18)
Missing, *n*	0	0
**Sex, *n* (%)**		
Female	211 (53)	967 (58)
Male	184 (47)	712 (42)
Missing, *n*	0	0
**Symptoms, *n* (%)**		
Cough	368 (93)	1452 (86)
Short breath	64 (16)	245 (15)
Sore throat	322 (82)	1441 (86)
Coryza	347 (88)	1462 (87)
Fever	336 (85)	1145 (68)
Malaise	373 (94)	1515 (90)
Myalgia	311 (79)	1061 (63)
Headache	297 (75)	1083 (65)
**BMI, *n* (%)**		
Normal	148 (37)	606 (36)
Obese	247 (63)	1073 (64)
Missing, *n*	0	0
**Chronic condition, *n* (%)**		
Presence of at least one chronic condition ^a^	114 (29)	495 (29)
**No chronic condition, *n* (%)**	281 (71)	1148 (70)
Missing, *n*	0	0
**GP consultation during the previous 365 days, *n* (%)**		
0–4	223 (56)	962 (57)
5+	172 (44)	717 (43)
**Hospitalization for any reasons during the previous 365 days, *n* (%)**		
Yes	11 (3)	81 (5)
No	384 (97)	1598 (96)
Missing, *n*	0	0
**Number of days between symptom onset and swabbing, *n* (%)**		
Max 2 days	255 (65)	933 (56)
3+ days	140 (35)	746 (44)
**Vaccination status, current season *n* (%)**		
Vaccinated	14 (4)	115 (7)
Unvaccinated	381 (96)	1564 (93)
Missing, *n*	0	0

^a^ The presence of a chronic condition is defined as reporting at least one of the following conditions: diabetes, heart disease, hypertension, lung disease, immunodeficiency, cancer, or renal disease.

**Table 2:** Vaccine effectiveness estimates against any influenza amongst patients aged 18 years old and over and in the target group for vaccination in the primary care-based VE study, Hungary, November 2024–May 2025.

Study Population	N	Cases	Cases Vaccinated	Controls	Controls Vaccinated	Adjusted VE *	95% CIs (Lower–Upper)	
All patients 18 years old and over	2074	395	14	1679	115	53	13	74
Target group for vaccination	782	145	12	637	96	52	7	75

* Models adjusted by age, sex, presence of at least one of the following chronic conditions (lung disease, heart disease, immunodeficiency and diabetes), and month of symptom onset.

**Table 3:** Vaccine effectiveness estimates against any influenza amongst patients aged 18 years old and over, according to time since vaccination, in the primary care-based VE study, Hungary, November 2024–May 2025.

Study Population	N	Cases	Cases Vaccinated	Controls	Controls Vaccinated	Adjusted VE *	95% CIs (Lower–Upper)	
All patients 18 years old and over & 14–89 days since vaccination.	2015	388	7	1627	63	63	17	84
All patients 18 years old and over & 90+ days since vaccination.	2004	388	7	1616	52	27	−67	68

* Models adjusted by age, sex, presence of at least one of the following chronic conditions (lung disease, heart disease, immunodeficiency and diabetes), and month of symptom onset, using Firth’s penalized logistic regression.

**Table 4:** Vaccine effectiveness estimates against any influenza amongst patients aged 18 years old and over, by seasonal vaccination status, in the primary care-based VE study, Hungary, November 2024–May 2025.

Study Population	N	Cases	Cases Vaccinated	Controls	Controls Vaccinated	Adjusted VE *	95% CIs (Lower–Upper)	
All patients 18 years old and over, current and prior season vaccinated.	1976	375	7	1601	70	56	1	80
All patients 18 years old and over, only prior season vaccinated.	2022	388	20	1634	103	5	−64	45

* Models adjusted by age, sex, presence of at least one of the following chronic conditions (lung disease, heart disease, immunodeficiency and diabetes), and month of symptom onset, using Firth’s penalized logistic regression.

**Table 5:** Influenza vaccine effectiveness estimates amongst patients aged 18 years old and over by influenza types in the primary care-based VE study, Hungary, November 2024–May 2025.

Study Population	N	Cases	Cases Vaccinated	Controls	Controls Vaccinated	Adjusted VE *	95% CIs (Lower–Upper)	
All patients 18 years old and over, influenza A	1937	258	13	1679	115	39	−16	68
All patients 18 years old and over, influenza B	1358	139	1	1219	88	80	2	96

* Models adjusted by age, sex, presence of at least one of the following chronic conditions (lung disease, heart disease, immunodeficiency and diabetes), and month of symptom onset, using Firth’s penalized logistic regression.

## Data Availability

The original contributions presented in the study are included in the article; further inquiries can be directed to the corresponding author.

## Supplementary Materials

The following supporting information can be downloaded at: https://www.mdpi.com/article/10.3390/vaccines14040342/s1, Supplementary Table S1: Vaccine effectiveness estimates against any influenza in the 18+ and in the target groups, model adjusted by rapid antigen test (RAT) usage, in the primary care-based IVE study, Hungary, November 2024–May 2025. Supplementary Table S2: Sensitivity analysis restricted to the predominant TIAV.
