# Supplementary material for: Primary Care-Based Estimates of Influenza Vaccine Effectiveness in Hungary, 2024/25
Source: Vaccines (Basel). 2026 Apr 13;14(4):342. doi: 10.3390/vaccines14040342 (PMC13120064; doi:10.3390/vaccines14040342)
Supplement: Supplementary file 1 [file vaccines-14-00342-s001.zip › vaccines-4228216-supplementary.pdf]

Primary Care-based Estimates of Influenza Vaccine Effectiveness in Hungary, 2024/25 - Supplementary material

Supplementary Table S1. Vaccine effectiveness estimates against any influenza amongst patients aged 18 and over, and in the target groups, model adjusted by rapid antigen test (RAT) usage, primary care-based influenza VE study, Hungary, November 2024–May 2025.

Study population	N	Cases	Cases vaccinated	Controls	Controls vaccinated	Adjusted VE*	95% CIs (Lower – Upper)	
All patients 18 years old and over	2074	395	14	1679	115	53	13	74
Target group	782	145	12	637	96	52	7	75

* Models adjusted by age, sex, presence of at least one of the following chronic conditions (lung disease, heart disease, immunodeficiency and diabetes), month of symptom onset and rapid antigen test (RAT) usage in the current illness episode.

Supplementary Table S2. Sensitivity analysis restricted to the predominant TIAV.

Study population	N	Cases	Cases vaccinated	Controls	Controls vaccinated	Adjusted VE*	95% CIs (Lower – Upper)	
All patients 18 years old and over	2068	395	14	1673	109	53	13	74
Target group	780	145	12	674	94	52	7	75
All patients 18 years old and over & 14-89 days since vaccination	2009	388	7	1621	57	65	20	84
All patients 18 years old and over & 90+ days since vaccination	2004	388	7	1616	52	30	-63	70

* Models adjusted by age, sex, presence of at least one of the following chronic conditions (lung disease, heart disease, immunodeficiency and diabetes), and month of symptom onset.
